# Atherosclerosis and Coenzyme Q_10_

**DOI:** 10.3390/ijms20205195

**Published:** 2019-10-20

**Authors:** Juan M. Suárez-Rivero, Carmen J. Pastor-Maldonado, Mario de la Mata, Marina Villanueva-Paz, Suleva Povea-Cabello, Mónica Álvarez-Córdoba, Irene Villalón-García, Alejandra Suárez-Carrillo, Marta Talaverón-Rey, Manuel Munuera, José A. Sánchez-Alcázar

**Affiliations:** Centro Andaluz de Biología del Desarrollo (CABD-CSIC-Universidad Pablo de Olavide), and Centro de Investigación Biomédica en Red: Enfermedades Raras, Instituto de Salud Carlos III, 41013 Sevilla, Spain; juasuariv@gmail.com (J.M.S.-R.); carmenj3b@gmail.com (C.J.P.-M.); mrdelamata@gmail.com (M.d.l.M.); marvp75@gmail.com (M.V.-P.); sulevapovea@gmail.com (S.P.-C.); monikalvarez11@hotmail.com (M.Á.-C.); villalon.irene@gmail.com (I.V.-G.); asuacar1@gmail.com (A.S.-C.); martatalrey@gmail.com (M.T.-R.); manolomunu@gmail.com (M.M.)

**Keywords:** atherosclerosis, ubiquinone, aging, coenzyme Q_10_

## Abstract

Atherosclerosis is the most common cause of cardiac deaths worldwide. Classically, atherosclerosis has been explained as a simple arterial lipid deposition with concomitant loss of vascular elasticity. Eventually, this condition can lead to consequent blood flow reduction through the affected vessel. However, numerous studies have demonstrated that more factors than lipid accumulation are involved in arterial damage at the cellular level, such as inflammation, autophagy impairment, mitochondrial dysfunction, and/or free-radical overproduction. In order to consider the correction of all of these pathological changes, new approaches in atherosclerosis treatment are necessary. Ubiquinone or coenzyme Q_10_ is a multifunctional molecule that could theoretically revert most of the cellular alterations found in atherosclerosis, such as cholesterol biosynthesis dysregulation, impaired autophagy flux and mitochondrial dysfunction thanks to its redox and signaling properties. In this review, we will show the latest advances in the knowledge of the relationships between coenzyme Q_10_ and atherosclerosis. In addition, as atherosclerosis phenotype is closely related to aging, it is reasonable to believe that coenzyme Q_10_ supplementation could be beneficial for both conditions.

## 1. Methods

For the purpose of this review, a systematic search strategy was developed to identify basic research works and clinical trials from January 1980 to October 2019 in MEDLINE (National Library of Medicine), Embase (Excerpta Medica database), Web of Science, Scopus, Google Scholar and the Cochrane Register of Controlled Trials (The Cochrane Collaboration). The terms “coenzyme Q_10_”, “ubiquinone”, “atherosclerosis”, “mitochondrial dysfunction”, “endothelial function”, “hypercholesterolemia”, “familial hypercholesterolemia”, “dyslipidemia”, “hypertension”, “metabolic syndrome”, “inflammation”, “inflammasome”, “endothelial function”, “aging”, “senescence”, “AMPK”, and “cardiovascular disease” were incorporated into an electronic search strategy. The authors reviewed all of the citations retrieved from the database search to identify recent and significant articles for this review.

## 2. Atherosclerosis: Old and New Approaches

Cardiovascular diseases (CVD) lead the cause of mortality worldwide, accounting for 16.7 million deaths every year [1], about one-third of total global deaths. Atherosclerosis, an inflammatory disorder of the vasculature, is the primary cause of CVD-related events, including myocardial infarction and stroke. Given the increase in the prevalence of risk factors, such as obesity and diabetes in developed countries, the global incidence of CVD is predicted to rise and impose a greater economic burden on the health-care services around the world.

The development of atherosclerosis is classically thought to be the result of dyslipidemia. Both high-density lipoprotein (HDL) and low-density lipoprotein (LDL) play critical roles in the transport of cholesterol and have been implicated in atherosclerosis [2]. Specifically, elevated levels of LDL and LDL-cholesterol (LDL-C) have been implicated in atherosclerosis progression [3]. In contrast, normal levels of HDL and HDL-cholesterol (HDL-C) are associated with a variety of antiatherogenic processes and reduced risk of CVD [2]. Therefore, classical strategies for treating atherosclerosis aimed at lowering LDL levels and increasing HDL levels in the blood.

The first stage of atherosclerosis is the internalization of cholesterol via circulating LDL in the arterial intima, promoting endothelial activation/dysfunction. The vascular endothelium is a semipermeable barrier that controls the diffusion of plasma molecules and regulates vascular tone, inflammation and prevents thrombus formation [4]; all these properties are altered in a dysfunctional endothelium. This infiltration of LDL into the extracellular matrix (ECM) stresses nearby cells and promotes circulating monocytes’ recruitment and attachment to the vascular endothelium. Monocyte recruitment from the bloodstream is probably the initial stage in the process of atherosclerotic plaque formation, activated by a regulated multistep process and mediated by chemoattractants, cell adhesion molecules and their receptors [5]. Once attached, they transmigrate into the sub-endothelial space where they are transformed into macrophages [6].

Moreover, alterations in the endothelial-related antithrombotic properties facilitate platelets adhesion and their activation in the dysfunctional area. Adhered platelets, in arrangement with dysfunctional endothelial cells, secrete chemotactic cytokines and growth factors, which stimulate migration, accumulation and proliferation of vascular smooth muscle cells (VSMC) and leukocytes in the intimal layer, enhancing plaque progression [7]. The roles of chemokines in atherosclerosis, particularly in the recruitment of monocytes, have been extensively reviewed [8,9].

LDLs retained in the ECM mainly by proteoglycans become targets for oxidative and enzymatic modifications. Then, oxidized LDLs (oxLDLs) enhance a series of pro-inflammatory reactions via different mediators perpetuating the activation, recruitment and transmigration of monocytes and other inflammatory cells across the endothelial layer into the intima. The attracted macrophages scavenge oxLDLs, become laden with lipids, and eventually converted into foam cells (macrophages full of lipid drops) [10,11]. Other cell types, such as endothelial (ECs) and VSMCs, can also become foam cells [12]. Several groups have reported that targeting to foam cells could effectively ameliorate atherosclerotic progression [13,14,15,16].

In the early steps of atherosclerosis, accumulation of foam cells evolves into fatty streaks (irregular yellow-white discoloration on the luminal surface of arteries), the first grossly visible lesion in the development of atherosclerosis [17]. A further complication of the lesion occurs when foam cells release growth factors and cytokines, which stimulate VSMC migration from the media into the intima where they divide and produce extracellular matrix components, such as collagen and contribute to the formation of a fibrous cap infiltrated with inflammatory cells (macrophages and lymphocytes) [18]. As the atheroma progresses, the number of VSMCs decreases and foam cells undergo apoptosis releasing cholesterol, pro-thrombotic molecules and active metalloproteases that degrade the fibrous cap of atherosclerotic plaques, increasing the susceptibility of plaque to rupture [19]. Plaque disruption and the subsequent exposure to thrombogenic substrates initiate both platelet adhesion/activation and aggregation on the exposed vascular surface and the activation of the coagulation cascade, leading to thrombus formation and clinical manifestations of the atherosclerotic disease, such as acute myocardial infarction or sudden death [20].

## 3. Atherosclerosis Treatment

Due to the high relevance of cholesterol in atherosclerosis, almost all therapeutic strategies are focused on reducing cholesterol blood levels [21] (Table 1). The most common and effective ones are statins, inhibitors of the 3-hydroxy-3-methyl-glutaryl-coenzyme A reductase (HMGCR) which is the rate-controlling enzyme of the mevalonate pathway. Interestingly, this pathway produces cholesterol and other isoprenoids, such as coenzyme Q_10_ (CoQ) [22]. In addition to statins, other therapeutic strategies have been developed, for instance using inhibitors of the N-terminal Niemann-Pick C1-like protein 1 (NPC1L1) receptor, which is located on the luminal surface of enterocytes and is responsible for cholesterol uptake [23]. Recently, innovative therapies using not only chemical drugs, but also monoclonal antibodies against certain proteins, such as the proprotein convertase subtilisin/kexin type 9 (PCSK9) have been utilized with promising results. In humans, PCSK9 naturally occurs and interacts with the LDL receptor (LDL-R) to stimulate its degradation and prevent its recycling to the cell membrane. Inhibition of PCSK9, therefore, results in an increased presence of LDL-R in the plasma membrane able of binding and internalizing LDL particles [24]. To date, two other therapeutic options have been approved: Microsomal transfer proteins (MTP) inhibitors and antisense oligonucleotide (ASO) against ApoB-100. MTP inhibitors lead to a disruption of the lipoprotein synthesis in the intestines and liver, yielding a reduction in LDL and triglycerides [25]. ASO drug also inhibits the synthesis of ApoB-100, a major constituent of LDL particles, at the mRNA level [26].

However, and despite these therapeutic options, the risk of atherothrombotic complications remains high, causing millions of deaths around the world each year. The notion of atherosclerosis has gradually evolved from a pure cholesterol accumulation in the great arteries to a lipid-driven, chronic, low-grade inflammatory disease of the arterial wall [35]. Over time, anti-inflammatory strategies are increasingly being considered as an attractive strategy to further reduce the residual risk of atherosclerotic cardiovascular disease [36]. It is known that statins are the most efficient therapeutic drugs against these diseases, since they both reduce the levels of atherogenic lipoproteins and prevent major cardiovascular events. Additionally, statins have anti-inflammatory effects independent of LDL reduction that may contribute to the treatment of atherogenesis and other cardiovascular diseases [37].

The notion that atherosclerosis was a fundamentally inflammatory disease began to gain popularity at the end of the twentieth century by the pioneering reports of Russel Ross and Peter Libby [38,39,40]. The formation and progression of atherosclerotic plaques is, thus, a consequence of focal ECs injury and inflammation [41]. However, as more of the underlying molecular mechanisms and signaling pathways controlling atherosclerosis are unveiled, it becomes more evident that atherosclerosis is the result of a variety of independent pathways and their complex interactions [42]. These pathways involve a multitude of various cell types, including monocytes, macrophages and bone marrow-derived progenitor cells, as well as cellular and subcellular processes, including mitochondrial dysfunction, autophagy alterations, inflammasome activation, early cellular senescence and cell death.

At present, atherosclerosis is considered an inflammatory disease [43], since the immune system and inflammatory response play a crucial role in its development and progression [44]. Atherosclerosis is characterized not only by the atherosclerotic plaque formation, but also by the accumulation of monocytes/macrophages, smooth muscle cells and lymphocytes within the arterial wall. Lipid uptake by monocytes/macrophages promotes their differentiation into large, lipid-laden, inflammatory foam cells in the vessel wall. The accumulation of inflammatory cells leads to the production of reactive oxygen species (ROS) and cytokines [45]. For these reasons, the former belief that the development of the atherosclerotic lesion solely depends on lipid deposition has been replaced by the current concept that activation of immune and inflammatory responses has a central role in plaque initiation and progression. Many anti-inflammatory strategies have emerged, thus, as potential treatments of atherosclerotic disease, in addition to the existing lipid-lowering therapies.

In combination with inflammation, it has been demonstrated that increased formation of ROS and/or altered oxygen utilization contributes to atherogenesis by superoxide production that mediates endothelial dysfunction and increases oxLDL levels. The small oxidized lipids that compose oxLDL, such as oxysterols, oxidized fatty acids and aldehydes, are potent inducers of ROS production [46]. ROS in the vascular wall is generated by enzymes, such as NADPH oxidase, xanthine oxidase and endothelial nitric oxide synthase [47]. Formation of intracellular ROS in the mitochondrial electron transport chain is controlled by antioxidant mechanisms. It has been shown that the increase of ROS generation by the mitochondria triggers cytochrome c release leading to caspase activation and apoptosis. The generation of large amounts of ROS can surpass the intracellular antioxidant defense, causing activation of neutrophils, protein modification, lipid peroxidation, mitochondrial alterations, and DNA damage, key factors for the initiation of atherosclerosis and the development of CVD [48].

All these new factors can be interconnected by mitochondrial dysfunction and inflammasome activation [49]. Mitochondrial dysfunction has been increasingly associated with the initiation and progression of atherosclerosis by elevating the production of ROS and mitochondrial oxidative stress damage [48]. Moreover, major precursors of atherosclerosis—hypercholesterolemia, hyperglycemia, hypertriglyceridemia, and even the process of aging—all induce mitochondrial dysfunction by affecting endothelial function, VSMC proliferation, macrophage activation and apoptosis [50]. On the other hand, mitochondria dysfunction also activates inflammasome by ROS production and releasing oxidized mitochondrial DNA (mtDNA) [51].

Inflammasomes are known as intracellular complexes which are able to convert pro-IL-1β and pro-IL-18 to mature forms through pro-caspase-1 cleavage and initiate the inflammatory response. Among them, nucleotide-binding domain, leucine-rich-containing family, pyrin domain-containing-3 (NLRP3) is a well-known inflammasome which has central roles in atherosclerosis [52,53,54]. It has been shown that NLRP3 inflammasome contributes to the progression of atherosclerosis via affecting a sequence of cellular and molecular targets by promoting local inflammatory responses and inducing pyroptosis [55]. Some pathogenic events, such as cholesterol crystals, oxidative stress, mitochondrial dysfunction, endoplasmic reticulum stress, and lysosome rupture, which are associated with atherosclerosis, could affect NLRP3 inflammasome activation [56]. Consequently, new therapies focusing on inflammasome activation and mitochondrial energizers should be considered as potential therapeutic strategies in atherosclerosis.

## 4. Coenzyme Q_10_: A Panacea?

**Figure 1:**
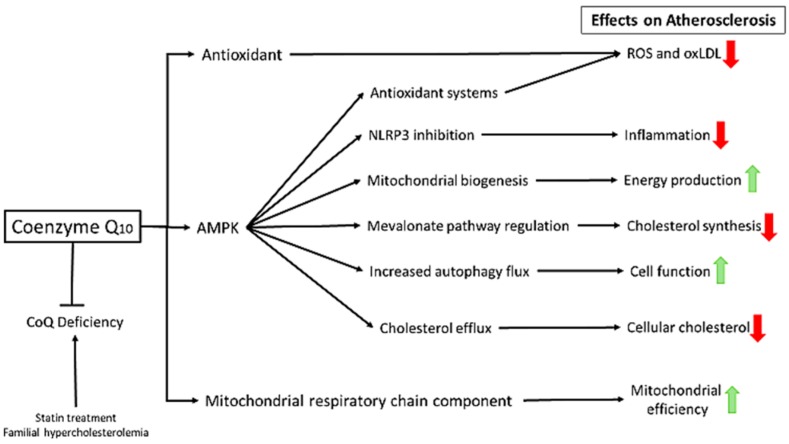
Coenzyme Q_10_ (CoQ) effects on atherosclerosis. CoQ is a lipophilic molecule composed by a benzoquinone ring conjugated to an isoprenoid chain of ten units in humans, which are the basis for its redox and lipophilic properties respectively. This molecule has a pleiotropic effect at several levels: Membrane antioxidant, cell signaling, gene expression and mitochondria function.

CoQ, also called ubiquinone, is known as a versatile molecule because of its participation in many cellular functions [57] (Figure 1). CoQ exists in both reduced and oxidized forms; conversion between these states allows it to transfer electrons to substrates and act as a cofactor of enzymatic reactions. CoQ is mainly required in the mitochondria as electron and proton carrier in the mitochondrial respiratory chain (MRC). The MRC, through oxidative phosphorylation, provides cells with the capacity to synthesize ATP, which is essential for cellular function. CoQ has several extramitochondrial activities, including AMPK activator, inflammasome regulator, mitophagy modulator, lipid-soluble antioxidant, prevention of membrane peroxidation, and regulation of the physicochemical properties of cell membranes [58]. Furthermore, CoQ has been shown to carry out epigenetic regulation in genes involved in cell signaling, intermediary metabolism, intracellular transport, transcription control, disease mutations, protein phosphorylation, and embryo development [59]. All these effects suggest that CoQ has an essential role in the modulation of gene expression, even though the underlying mechanisms are not yet fully understood [60]. In addition, CoQ may improve endothelial dysfunction, and can possibly enhance cardiac ATP production and cardiac output by exerting a positive inotropic effect upon the myocardium [61]. Interestingly, CoQ may also have a lowering effect on blood pressure [62,63,64].

Due to its function as a mitochondrial energizer, cell membrane antioxidant, anti-inflammatory capacity, ability to regulate gene expression and cardiovascular hemodynamics, CoQ has been proposed as an alternative/complementary treatment for cardiovascular disease in general and atherosclerosis in particular [65].

## 5. Familial Hypercholesterolemia and Atherosclerosis

**Figure 2:**
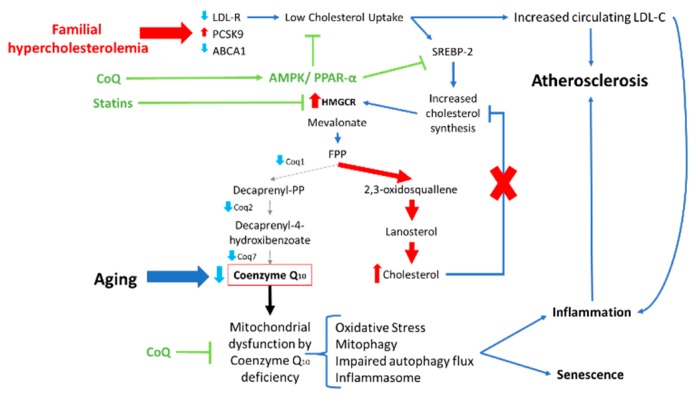
Working model of FH physiopathology and the effects of CoQ. First, LDL-C uptake is impaired, due to LDL-R mutations, provoking high levels of LDL-C in the blood. At the cellular level, poor extracellular cholesterol uptake will also result in dysregulated mevalonate pathway, which leads to cholesterol accumulation and secondary CoQ deficiency. Intracellular cholesterol accumulation is associated with SREBP-2 activation and increased HMGCR and cholesterogenic enzymes expression levels. As a consequence, CoQ biosynthesis, the other main sub-branch of the mevalonate pathway, is defective inducing mitochondrial dysfunction, oxidative stress, impaired autophagy flux and inflammasome activation. Interestingly, all these alterations are also involved in cell senescence. CoQ treatment can correct both altered mevalonate pathway and mitochondrial dysfunction in FH fibroblasts. CoQ causes a significant increased expression of LDL-R and ABCA1 accompanied by downregulation of PCSK9, as well as AMPK and PPAR-α activation. In addition, CoQ restores INSIG1 and INSIG2 expression levels (involved in intracellular cholesterol sensing) and allows the normal feedback inhibition of SREBP-2 activation when cholesterol levels are high. The beneficial effects of CoQ on mitochondrial function can also be attributed to CoQ biosynthetic pathway up-regulation. ABCA1, ATP-binding cassette transports A1; AMPK, AMP-activated protein kinase; CoQ, Coenzyme Q_10_; HMGCR, 3-Hydroxy-3-Methyl-Glutaryl-Coenzyme A Reductase; FPP, Farnesyl Pyrophosphate; LDL-C, Low Density Lipoprotein Cholesterol; LDL-R, Low Density Lipoprotein Receptor; PCSK9, Proprotein Convertase Subtilisin/Kexin type 9; PPAR-α, Peroxisome proliferator-activated receptors α; SREBP2, Sterol regulatory element-binding proteins.

Familial hypercholesterolemia (FH) is a common inherited disorder characterized by abnormally elevated serum levels of LDL-C from birth, which in time can lead to an early risk of CVD. Most cases are caused by autosomal dominant mutations in LDL-R, although mutations in other genes coding for proteins involved in cholesterol metabolism or LDL-R function and processing, such as APOB and PCSK9, can also be causative, although less frequently [66]. As FH is a genetic disease associated with premature atherosclerosis, patient-derived cells are an excellent model to study the cellular alterations responsible for the initiation/development of early atherosclerotic lesions (Figure 2).

According to a recent study of our group, FH fibroblasts bearing LDL-R mutations are unable to import extracellular cholesterol for its metabolism. Since cholesterol-uptake is impaired, FH fibroblasts synthesize such lipid endogenously in an uncontrolled manner which eventually leads to its accumulation [67]. The upregulation of cholesterol biosynthesis is directly related to an in increase HMCGR activity, which is controlled by lipoproteins’ binding to the LDL-R [68]. Bearing in mind that FH fibroblasts have dysfunctional LDL-R, it is not surprising that cholesterogenic enzymes are upregulated. As a consequence, the mevalonate pathway results to be shifted to an overproduction of cholesterol in detriment of CoQ biosynthesis, meaning this that these FH patients suffer from secondary CoQ deficiency. Both CoQ deficiency and cholesterol accumulation are associated with elevated ROS production, reduced ATP levels, low activity of mitochondrial respiratory complexes and mitochondrial depolarization. Indeed, FH fibroblasts showed increased elimination of dysfunctional mitochondria by mitophagy [69]. Interestingly, mitochondrial dysfunction was associated with inflammasome activation accompanied by increased production of IL-1β and IL-18.

Altogether, these findings suggest that mitochondrial dysfunction and CoQ deficiency could be partly responsible for the cellular pathophysiology of early atherosclerosis in FH by enabling increased production of free-radicals and inflammasome activation in the endothelium of blood vessels [69]. Aiming to ameliorate the conditions of FH patients, treatments targeted to both reducing intracellular cholesterol and raising CoQ levels should be considered. CoQ administration restored both cholesterol levels and mitochondrial function in cellular models of FH. Therefore, co-administration of the conventional statin treatment used for hypercholesterolemia patients in combination with CoQ seems to be a reasonable approach for FH therapy [67].

## 6. CoQ and Inflammation

From the point of view of inflammation, pro-inflammatory genes Tumor Necrosis Factor-α (TNF-α) and Interleukin-6 (IL-6) have been reported to be expressed at high levels and contribute to cardiac damage in hyperlipidemia, in addition to canonical inflammatory markers, such as IL-18 and IL-1β [70]. Several reports have shown that CoQ supplementation reduces most inflammatory parameters, including NLRP3 activation, mostly by restoring/enhancing mitochondrial function [71,72]. Arterial damage induced by atherosclerosis is usually associated with an increase number of macrophage-derived foam cells and the release of cytokines that recruit more macrophages to lesions that increase lipid deposition. Several studies have demonstrated that CoQ reduces macrophage accumulation, foam cell formation and lipid accumulation [73,74]. Moreover, one strategy that has been extensively studied related with the macrophage’s role in atherosclerosis, identified new ways to increase the removal of an excess of cholesterol from peripheral cells and lesions through the reverse cholesterol transport (RCT). This pathway is mediated by a number of lipid and cholesterol transporters, including the widely-studied cholesterol efflux proteins, ATP-binding cassette transports A1 and G1 (ABCA1 and ABCG1). CoQ seems to promote the macrophage RCT through a specifically microRNA-ABCG1 interaction, thus, contributing to prevent atherosclerosis progression [75].

CoQ is known to be a potent activator of AMPK [76], and many of its effects can be explained through this pathway. AMPK signaling pathways are involved in various physiological processes, such as metabolism, cytoskeleton reorganization, transcriptional control, apoptosis and autophagy. AMPK plays a critical role in the development of atherosclerosis via regulation of carbohydrate and lipid metabolism, as well as modulating the function of vascular smooth muscle cells, endothelial cells and immune cells. Thus, dysregulation of autophagy and reduced AMPK activity are associated with atherogenesis by increasing ROS and inflammatory cytokines production in the endothelium [77,78]. AMPK and autophagy generally promote cholesterol metabolism and attenuate the development of atherosclerosis via regulation of the expression of cholesterol transport-related proteins. Accumulating evidence demonstrate that autophagy in macrophages plays an important role in inhibiting inflammation and apoptosis, and in promoting efferocytosis and cholesterol efflux [79]. Furthermore, AMPK activation decreased the release of pro-inflammatory cytokines and increased the expression of the anti-inflammatory cytokine IL-10 in macrophages and the endothelium [80].

In endothelial cells, AMPK triggers numerous advantageous pathways, such as autophagy, mitochondrial biogenesis or antioxidants enzymes to minimize oxidative stress and redox imbalance which promotes endothelial dysfunction. Nitric oxide (NO) activates AMPK that also increases NO release via the phosphorylation of endothelial nitric oxide synthase, suggesting positive feedback [81]. Decreased NO bioavailability is associated with the increase of ROS production in vessel walls [82]. Moreover, enhanced endoplasmic reticulum stress and atherosclerosis in vivo result from decreased AMPK activity [83]. AMPK activation in the endothelium correlated with the function of VSMCs, especially with vasorelaxation via increased NO production. On the other hand, the vasoconstriction function of VSMCs primarily coordinates various physiological and pathological stimuli. Vascular inflammation, foam cells formation and the instability of atherosclerotic plaque are partially attributed to VSMCs proliferation and migration. Furthermore, AMPK activation in VSMCs induced vasodilatation and attenuated smooth muscle contraction [84]. Finally, defective autophagy in VSMCs accelerates senescence via the accumulation of sequestosomes and the promotion of neointima formation after injury and dietary-induced atherosclerosis [85]. Promotion of appropriate autophagy in VSMCs may effectively ameliorate atherosclerosis [85]. Overall, AMPK signaling has obvious implications in cardiovascular health and disease.

## 7. CoQ and Statin Myopathy

In general, statins are well-tolerated in most adults, with few serious adverse effects. However, they are frequently associated with mild muscle damage, known as statin-associated muscle symptoms (SAMS) which include myalgia, weakness and cramps. SAMS are more prevalent in women and older adults, although data do not always support this hypothesis [86]. Muscle side effects are a clinical concern because they reduce muscle strength, physical activity, quality of life, medication compliance, and ability to perform daily activities, and eventually result in preventable cardiac events [87].

Several researchers have argued that lipid-lowering per se may cause SAMS [88], but an alternative and more likely explanation is that inhibition of HMGCR reduces the formation of isoprenoids farnesyl pyrophosphate and geranylgeranyl pyrophosphate, resulting in reduced prenylation of small GTPase proteins involved in cell growth and maintenance [89]. Moreover, statin treatment also results in decreased formation of CoQ. Thus, much of SAMS may be caused by depletion of muscle levels of CoQ and resultant impairment of mitochondrial function [90,91,92]. The underlying mechanisms of statin myopathy were recently reviewed by the European Atherosclerosis Society Consensus Panel [93]. Vladutiu found that muscle levels of CoQ in patients with statin myopathy were 3 to 4 SDs below normal [94]. This effect is directly related to the potency of statins, although their lipophilicity may aggravate the problem, with a theoretical advantage of the most hydrophilic ones, such as rosuvastatin and pravastatin. Thus, for a given reduction in LDL cholesterol, rosuvastatin increased plasma levels of creatine kinase, a marker of muscle damage, less than other statins [95]. CoQ supplementation has commonly been thought to prevent statins side effects. Half of the multiple trials that have evaluated the impact of CoQ supplementation on SAMS present a beneficial outcome, whereas, the rest show no effect [86,96,97]. Nonetheless, CoQ administration remains a common therapy for the treatment of SAMS among physicians [98]. However, the doses required may need to be higher (200–400 mg twice a day) than in most of the conducted clinical trials [99].

## 8. CoQ Supplementation

The potential benefits of CoQ oral intake have led to an extensive interest in its use as a dietary supplement or as a drug. As a lipophilic substance, CoQ follows the same absorption process as that of lipids in the gastrointestinal tract. Thus, absorption of CoQ is enhanced in the presence of lipids. Following absorption, CoQ is incorporated into chylomicrons and is transported to the systemic circulation via the lymphatic system [100]. In plasma, CoQ is mainly carried by lipoproteins, mostly in LDL particles where it is predominantly found in its reduced form [100,101]. Determination of CoQ levels in the blood may be useful for assessing its content in the body and treatment adherence.

In general, CoQ has a remarkable safety profile that shows a low rate of adverse events [102,103,104,105,106]. However, some clinical trials found that CoQ treatment may produce nausea, heartburn, upset stomach or related gastrointestinal effects [107]. No adverse impacts other than these mild and transient gastrointestinal effects have been reported. A possible indirect adverse effect of oral CoQ ingestion would be a decreased endogenous biosynthesis and decreased blood and tissue levels resulting in a “rebound” deficiency if the oral supply should be discontinued. However, it has been shown that exogenous supplementation of the quinone did not influence the endogenous biosynthesis, and there was no accumulation in plasma or tissues after cessation of supplementation [103].

Apart from the diet, the body mostly relies on the endogenous synthesis of this coenzyme [108]. Therefore, CoQ deficiency is not expected to occur in healthy individuals because endogenous production is usually sufficient. Nevertheless, due to the small incorporation of CoQ from the diet, supplementation is the easiest way to increase CoQ levels to meet clinical requirements. However, CoQ supplementation presents several challenges mainly derived from its poor bioavailability. Exogenous CoQ is taken up from the intestine into chylomicrons, and hence, to the circulation with a range of between 2% and 4% of the total uptake, due to its poor solubility. In blood, the maximum concentration (C_max_) reached was 8,7 μM after large doses of 3600 mg/day [109]. Variables that affect absorption include the type of formulation, the dose administered, the dosing interval, whether the supplement is taken with or without food and with what kind of food. In fact, CoQ absorption could be saturated in a high single daily dose reducing its total bioavailability. In contrast, multiple low daily doses could improve its uptake [110].

The development of CoQ analogues, mostly for neurodegenerative and mitochondrial diseases, with a similar effect, but with a better bioavailability, is trending. Idebenone is a quinone developed molecule with increased water solubility. Idebenone has shown antioxidant activity, as well as the ability to inhibit lipid peroxidation in isolated brain mitochondria, synaptosomes, and cells. Moreover, it is thought to transfer electrons directly to complex III, thereby circumventing complex I and restoring cellular energy generation [111]. EPI-743 is a CoQ/vitamin E analogue developed for the treatment of inherited mitochondrial diseases [112,113]. EPI-743 is a para-benzoquinone targeting the repletion of reduced intracellular glutathione. EPI-743 is one thousand- to ten thousand-fold more potent than CoQ or idebenone in reducing oxidative stress in cell models [114].

Another approximation for increasing CoQ bioavailability implies using chemical combinations. For example, MitoQ is a CoQ molecule attached to a lipophilic triphenylphosphonium cation (TPP+), which allows CoQ to target mitochondrial matrix specifically. When the quinol form of MitoQ acts as an antioxidant, it is oxidized to the quinone form, which is then rapidly re-reduced by complex II, restoring its antioxidant efficacy [115]. However, MitoQ cannot restore respiration in mitochondria lacking CoQ because the reduced quinol form of MitoQ is not oxidized by complex III, and therefore, cannot act as an electron carrier [116]. However, the modification of CoQ could make it more bioavailable or a better antioxidant, but it would deprive it of lipophilic activity and probably its interaction with certain proteins and factors.

Some alternatives have surged like solid dispersion systems [117], nanoparticles [118], cyclodextrin inclusion compounds and microcapsules [119]. However, one of the most promising approaches has been the preparation of nano-liposomes with long-circulating elements that improve the stability, prolong circulation times and increase the bioavailability of CoQ [120].

## 9. Looking behind Atherosclerosis: Aging

Aging is the dominant risk factor for the formation of clinically significant atherosclerotic lesions; however, the greatest impact of aging on the disease is not explained by changes in traditional risk factors, such as lack of physical activity, smoking, hypertension, hyperlipidemia or diabetes mellitus [121]. Therefore, aging is considered as an independent risk factor for its development. Atherosclerosis is associated with premature biological aging, since atherosclerotic plaques show evidence of cellular senescence, which is characterized by elevated DNA damage, epigenetic modifications, telomere shortening and dysfunction, irreversible growth arrest and eventually apoptosis [122]. The interconnection between both processes could be explained by the increase in critical factors related to aging, such as inflammation [123], and reduced CoQ biosynthesis associated with mitochondrial dysfunction [59], both of them also participating in atherosclerosis progression [48,124].

Human aging is a normal multifactorial process resulting from the interaction of genetic and environmental factors [125]. A common hypothesis to explain some of the age-related pathophysiological and degenerative diseases is the oxidative imbalance between the production of ROS and antioxidant mechanisms, such as superoxide dismutase, catalase, glutathione peroxidase, ascorbic acid, tocopherol, glutathione, and CoQ, leading to a state of oxidative stress [126]. As mitochondria are the main source of ROS production through OXPHOS, this organelle is the major target of ROS damage. Mitochondrial DNA is particularly vulnerable, with a high mutation rate and limited mtDNA repair mechanisms [127]. All these postulates configure the so-called oxidative stress (or free-radical) theory of aging [128]. Although the accumulation of ROS has a major effect on DNA (strand breaks, oxidation of bases, damage in sites coding for MRC proteins), other structures of the cells are also damaged, including lipids, membranes and proteins among others. There is evidence that impaired mitochondrial machinery produces more oxidative stress and ROS, resulting in a vicious cycle [129,130]. The overproduction and accumulation of these oxygen species could lead to chronic inflammation, a frequent aging-related problem [131]. All of these features can be detected in cells from atherosclerotic plaques, which show additional characteristics of cell senescence [132].

As the normal function in mitochondrial electron transport depends on CoQ levels, where it has been observed that the main electron leaks occur between the complex I and II to complex III [133], CoQ levels decline with aging may explain the postulated increase in ROS production with advanced age. Furthermore, multiple studies have demonstrated a key role for mitochondria or mitochondrial function in inflammasomes activation [134,135]. Mitochondrial dysfunction or damage cause the following individually or simultaneously: Mitochondrial ROS (mtROS) production, the release of mtDNA, aberrant calcium mobilization, reduction in cytoplasmic levels of NAD+, potassium (K+) efflux, as well as extracellular ATP. These mitochondrial-related changes have been shown to be involved in NLRP3 inflammasome activation [136]. Thus, aging-related reduced CoQ levels may contribute to inflammation, and CoQ supplementation may prevent its progression [137]. In addition, a reduced CoQ content promotes mitochondrial permeability transition and bioenergetic dysfunction leading to premature aging of human dermal fibroblasts in vitro [138]. Furthermore, supplementation with reduced CoQ (Ubiquinol) prevents senescence and dysfunction caused by oxidative stress in vascular endothelial cells suggesting that it could delay vascular aging [139]. Both vascular aging and cellular senescence are associated with increased expression of proinflammatory cytokines and adhesion molecules, further promoting inflammation and atherosclerotic lesions [140].

As advanced atherosclerosis is likely to manifest irreversible changes, prevention of accelerated cell aging becomes a major therapeutic strategy. It is not yet well known whether low CoQ content is an effect of aging, perhaps associated with the progressive decline of mitochondrial electron transport function, or a contributing cause to the aging process. Understanding the mechanisms that trigger such changes is, therefore, crucial for both the prevention and the development of treatment options for atherosclerosis and other age-related diseases.

## 10. Future Research Directions

There is still much to be learned about the potential benefits of CoQ supplementation in atherosclerosis. First, the knowledge of cellular alterations in the arterial wall may shed light on the involvement of CoQ deficiency in the onset of the local inflammatory process leading to the formation of the arteriosclerotic plaque. Second, deeper studies on the relationship between LDL-R expression and cellular cholesterol uptake and its effect on intracellular metabolic pathways, such as the mevalonate route and its sub-branches will provide more information about the origin of mitochondrial dysfunction in early atherosclerosis. Finally, respect to the beneficial effects of CoQ supplementation in atherosclerosis initiation and prevention there remains a lack of long-term studies with larger amounts of subjects using better formulations capable of reaching effective CoQ concentrations in blood and tissues.

## Figures and Tables

**Table 1:** Main approaches in atherosclerosis treatment.

Strategy	Drug	Drawback	Source
To block endogenous cholesterol biosynthesis	Statins	Diabetes Myopathies	[27,28]
To block cholesterol intestinal absorption	Ezetimibe	Vitamin absorption inhibition	[29,30]
To reduce LDL receptor degradation	Evolocumab Alirocumab	Immunological response in rare cases High price	[31,32]
To disrupt LDL synthesis	Lomitapide Mipormesren	Hepatotoxicity Diarrhea Increased cardiovascular risk	[33,34]

## Abbreviations

ABCA1	ATP-binding cassette transport A1
ABCG1	ATP-binding cassette transport G1
AMPK	AMP-activated protein kinase
ASO	Antisense Oligonucleotide
CoQ	Coenzyme Q_10_
CVD	Cardiovascular diseases
EC	Endothelial Cell
FPP	Farnesyl Pyrophosphate
HDL	High Density Lipoprotein
HMGCR	3-Hydroxy-3-Methyl-Glutaryl-Coenzyme A Reductase
IL	Interleukin
LDL	Low Density Lipoprotein
LDL-R	Low Density Lipoprotein Receptor
NLRP3	Nucleotide-binding domain leucine-rich-containing family pyrin domain-containing-3
mtDNA	Mitochondrial DNA
MRC	Mitochondrial Respiratory Chain
MRC	Mitochondrial Respiratory Chain
mtROS	Mitochondrial ROS
MTP	Microsomal Transfer Proteins
NLRP3	Nucleotide-binding domain leucine-rich-containing family pyrin domain-containing-3
NO	Nitric Oxid
NPC1L1	N-terminal Niemann-Pick C1-like protein 1
oxLDL	Oxidized LDL
OXPHOS	Mitochondrial Oxidative Phosphorylation System
PCSK9	Proprotein Convertase Subtilisin/Kexin type 9
PPAR-α	Peroxisome proliferator-activated receptor-alpha
ROS	Reactive Oxygen Species
RCT	Reverse Cholesterol Transport
SAMS	Statin-Associated Muscle Symptoms
SREBP-2	Sterol regulatory element-binding proteins 2
TNF-α	Tumor Necrosis Factor-α
VSMCs	Vascular Smooth Muscle Cells
